# Primary Periumbilical Endometriosis Mimicking Umbilical Hernia in a 37-Year-Old Woman: A Report of a Rare Case

**DOI:** 10.7759/cureus.108541

**Published:** 2026-05-09

**Authors:** Sarmistha Gupta, Rajalakshmi Srinivasan

**Affiliations:** 1 Radiology, Medcare Royal Speciality Hospital, Dubai, ARE; 2 Obstetrics and Gynaecology, Medcare Royal Speciality Hospital, Dubai, ARE

**Keywords:** endometriosis, fibroid, hernia, menorrhagia, myomectomy, umbilicus

## Abstract

This case report describes a rare presentation of primary periumbilical endometriosis, also known as Villar’s nodule, highlighting the diagnostic difficulties and the importance of maintaining clinical suspicion. A 37‑year‑old woman presented with a one‑year history of cyclical periumbilical pain along with severe menorrhagia and dysmenorrhea. Initial ultrasonography suggested an umbilical hernia, but subsequent imaging, including repeat ultrasound and contrast‑enhanced MRI, revealed subcutaneous periumbilical nodules without evidence of intraperitoneal extension or associated pelvic endometriosis. An incidental intramural uterine fibroid was also noted. The patient underwent surgical excision of the periumbilical nodules along with myomectomy. Histopathological examination confirmed the diagnosis of endometriosis. Her postoperative recovery was uncomplicated, and she remained asymptomatic at the six‑month follow‑up. This case underscores the need to consider primary periumbilical endometriosis in women presenting with cyclical umbilical pain and demonstrates that early diagnosis and complete surgical excision can lead to favorable clinical outcomes.

## Introduction

Endometriosis is a chronic and progressive condition characterized by the presence of functional endometrial glands and stroma located outside the uterine cavity [1]. It is a benign, oestrogen-dependent pathological condition that affects approximately 6-15% of women during their reproductive years [2]. According to the World Health Organization (WHO) estimates, approximately 10% of women of reproductive age, around 190 million globally, are affected by endometriosis [3].

This condition most commonly involves pelvic organs such as the ovaries and uterine ligaments, but in rare cases, about 0.5-1%, it occurs outside the uterine cavity. These extragenital manifestations can involve sites like the intestinal tract, lungs, thoracic region, and surgical scars. Among these, primary umbilical endometriosis is the rarest form, characterized by ectopic endometrial tissue within the umbilicus [1,4].

Umbilical endometriosis may present as either primary (spontaneous) or secondary (following surgical manipulation). The primary form, which occurs in the absence of prior abdominal or umbilical surgery, is exceptionally uncommon. Pathogenesis remains debated, with hypotheses including retrograde menstruation with coelomic metaplasia, lymphatic or hematogenous spread, and coelomic transformation of multipotent cells [4].

Clinically, patients often present with an umbilical nodule that appears red, purple, or black, and shows cyclical pain, swelling, discoloration, and occasionally bleeding in sync with menstrual cycles. The symptoms differ among individuals and may include dysmenorrhea, dyspareunia, dyschezia, and dysuria. It affects 70% of women with chronic pelvic pain. Pelvic pain frequently begins before menstruation, though some cases remain asymptomatic and are detected only during infertility evaluation [5,6].

Differential diagnoses often include umbilical hernia, granuloma, melanoma, or subcutaneous tumors [4]. Diagnosis is challenging because umbilical endometriosis often mimics conditions such as hernias, cysts, or tumors, and imaging lacks specific features [7]. While ultrasound and MRI can help localize subcutaneous lesions and assess vascularity, definitive diagnosis requires surgical excision followed by histopathological confirmation [4,8].

Here, we present the case of a 37‑year‑old woman presenting with primary periumbilical endometriosis manifested by cyclical pain and nodules without evidence of pelvic involvement, alongside an incidental intramural fibroid. We discuss diagnostic challenges, imaging findings, and surgical management, providing insight to guide clinicians in recognizing and treating this rare extragenital presentation. This case report has been reported in line with SCARE (Surgical CAse REport) criteria [9].

## Case presentation

Clinical history

A 37-year-old woman, para 1, presented with severe menorrhagia, dysmenorrhea, and cyclic pain localized to the umbilicus during the immediate premenstrual and menstrual phases for the preceding year. Her obstetric history revealed a lower segment cesarean section (LSCS) performed six years earlier through a Pfannenstiel incision. There was no prior history of endometriosis, and notably, her current symptoms started only one year ago.

Investigations

**Figure 1:**
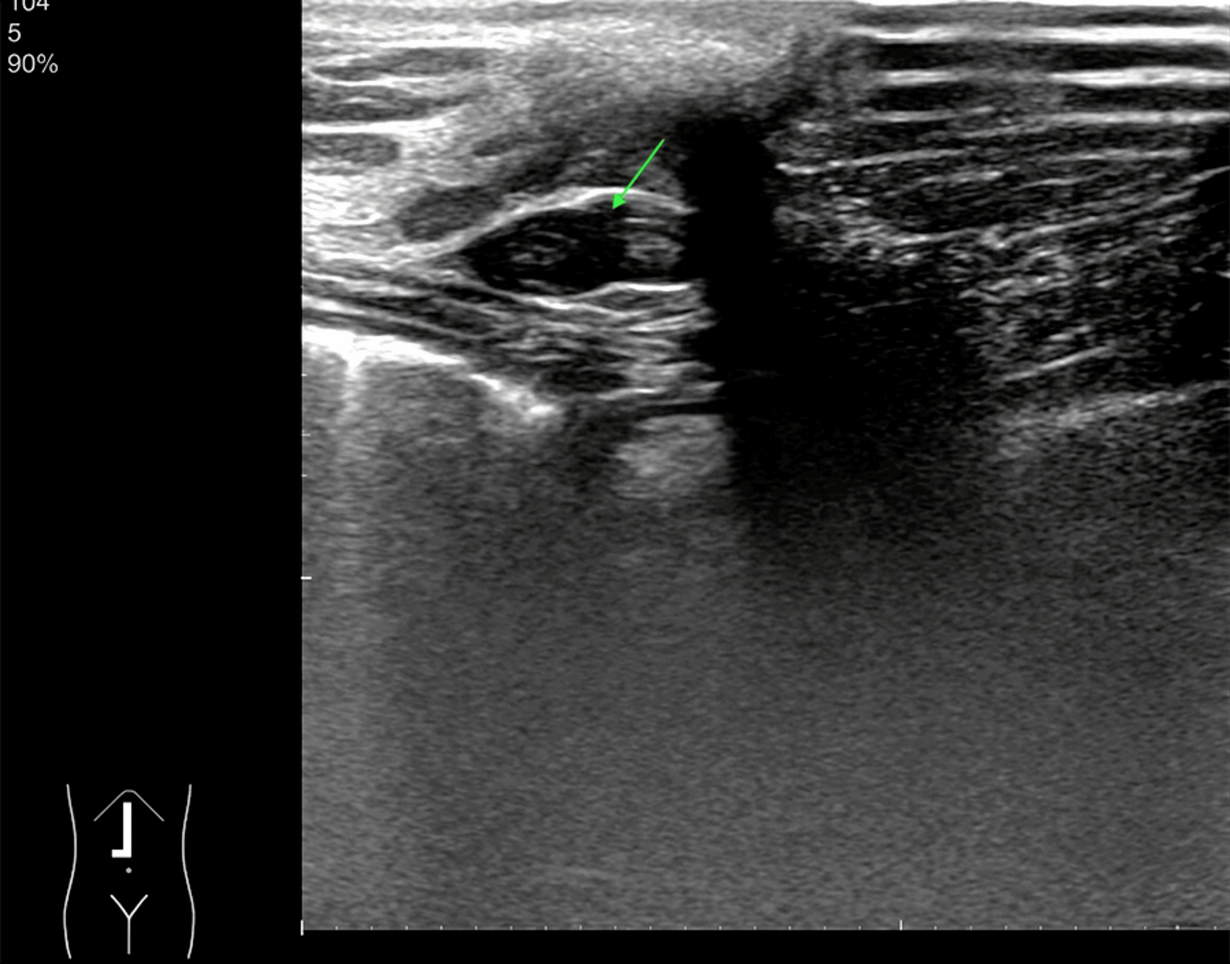
Ultrasound image showing a well-defined oval solid-cystic hypoechoic subcutaneous nodule (arrow) measuring 8.9 × 5 × 5.3 mm in the immediate supraumbilical region along the midline of the abdomen.

An ultrasound performed at an outside facility a month prior to this presentation suggested an umbilical hernia containing omentum. However, a repeat abdominal ultrasound at our hospital revealed two solid-cystic hypoechoic subcutaneous nodules with well‑defined margins, measuring 8.9 x 5 x 5.3 mm in the immediate supraumbilical region, along the midline of the abdomen. The lesions were superficial, located within the subcutaneous fat, and were painful during the perimenstrual phase (Figure 1).

There was no facial defect in the underlying abdominal wall beneath the lesion, indicating that it was not a hernia. This was further confirmed by dynamic assessment using the Valsalva manoeuvre and cough impulse, which revealed no to‑and‑fro movement of the lesion, thereby excluding the possibility of an umbilical hernia.

**Figure 2:**
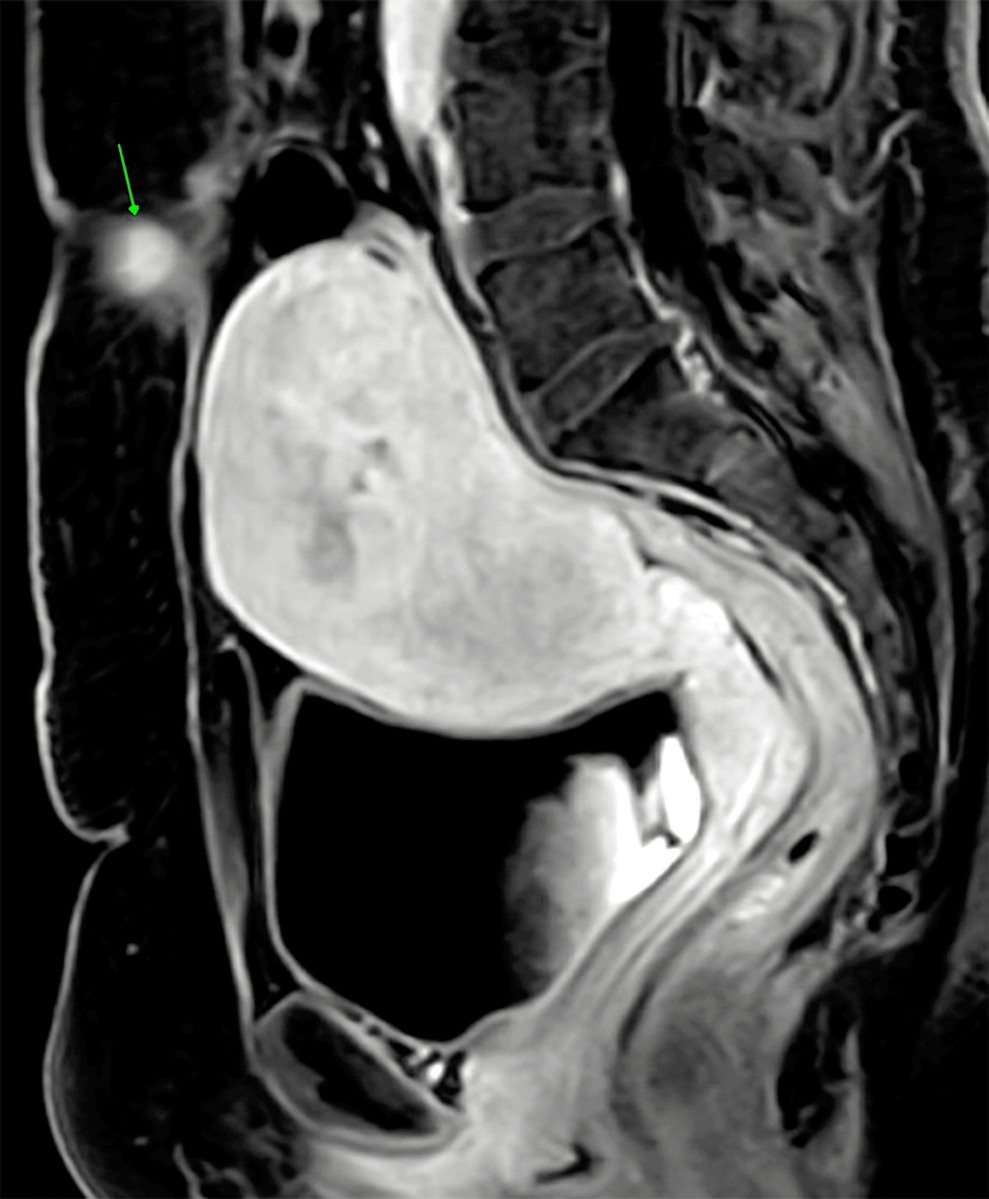
Sagittal T1 FS postcontrast MRI sequence shows enhancing subcutaneous solid lesion (arrow) within the umbilicus. No intraperitoneal extension is seen. Anteverted bulky uterus is seen due to an intramural leiomyoma

**Figure 3:**
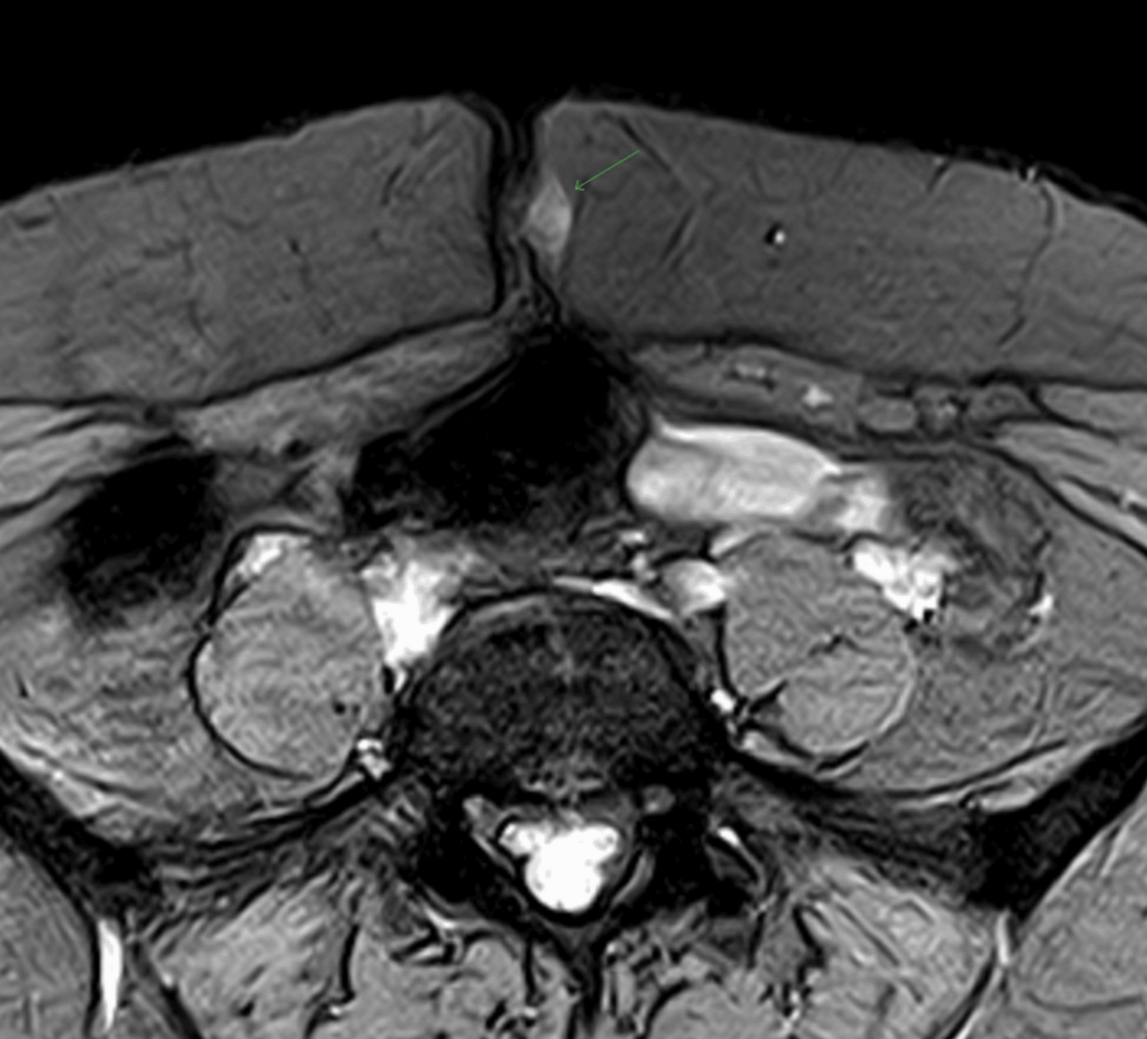
Axial T2 FS MRI image showing a hyperintense signal intensity subcutaneous lesion (arrow) within the umbilicus. No intraperitoneal extension is seen.

Based on the cyclical nature of pain and imaging findings, endometriosis was suspected, and an MRI with contrast was recommended for confirmation. The MRI demonstrated two oval solid lesions located superficially within the subcutaneous fat of the umbilical region, with no communication with the peritoneal cavity or intraperitoneal extension (Figures 2, 3).

The first lesion measured 1.5 × 1.9 × 0.8 cm, and the second measured 1.3 × 1.5 × 0.7 cm. Both lesions showed intermediate signal intensity on T1-weighted images and hyperintense signal on T2 fat-suppressed sequences, with homogeneous post-contrast enhancement. Gradient sequences showed a few tiny foci of blooming, suggestive of hemorrhagic components. There was no evidence of intraperitoneal extension. Importantly, no endometriotic deposits were identified in the ovaries or elsewhere in the abdomen and pelvis.

An incidental finding of an intramural fibroid measuring 5.7 × 5.5 × 5.2 cm was noted at the uterine fundus, indenting the endometrium.

Diagnosis

Based on clinical history and imaging, a diagnosis of primary periumbilical endometriosis with an associated intramural fibroid was made.

Management

The patient underwent surgical management, including laparoscopic myomectomy along with laparoscopic excision of the umbilical nodules. Surgery was performed one day prior to menstruation, which facilitated palpation and intraoperative identification of the lump due to its characteristic bluish appearance. Intraoperatively, three closely clustered nodules were identified and were removed together with a wide margin of about 1 cm. The nodules were close to, but not involving, the umbilicus; hence, umbilical preservation was achieved, with the incision placed along the lower margin of the umbilicus. No pelvic endometriosis was identified intraoperatively.

**Figure 4:**
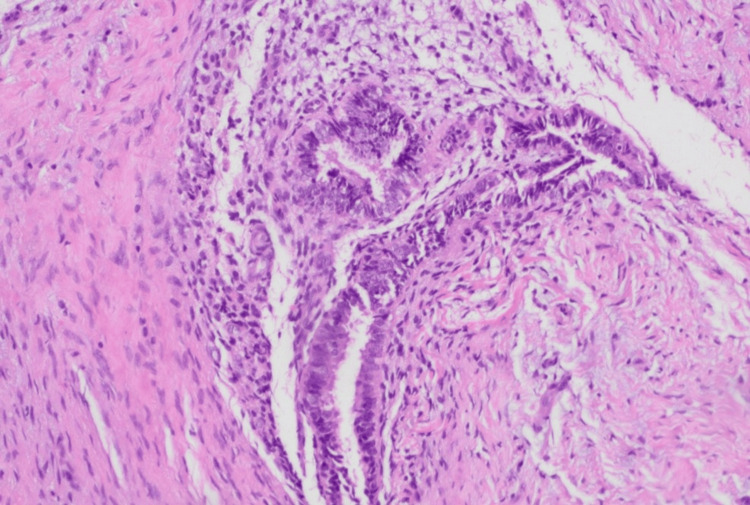
Histopathology showing fragments of muscle tissue (H&E stain) with multiple foci of endometrial glands and stroma embedded within (×40).

Histopathological examination of the excised umbilical lesion showed a fragment of adipose tissue along with hemorrhage and muscle tissue showing multiple foci of endometrial gland and stroma embedded within it, with no evidence of atypia, confirming the diagnosis of umbilical endometriosis (Figure 4).

Outcome

The patient had an uneventful postoperative recovery with no complications. Pain and menorrhagia resolved completely following surgical excision of the umbilical nodules and myomectomy.

Follow-up

At the three-month follow-up, the patient remained asymptomatic with no evidence of recurrence of umbilical endometriosis or fibroid-related symptoms. She reported a significant improvement in overall quality of life.

## Discussion

Umbilical endometriosis is a rare manifestation of extragenital endometriosis, with the majority of cases representing primary disease rather than secondary implantation. Approximately 70-75% of reported cases are spontaneous in origin and are commonly referred to as Villar’s nodule, first described by Villar in 1886, and represents a small subset of cutaneous endometriosis and is believed to arise through mechanisms such as lymphatic or hematogenous dissemination of endometrial cells or metaplastic transformation [2,5,10,11]. Because of its low prevalence and nonspecific clinical presentation, umbilical endometriosis is often misdiagnosed as more common umbilical conditions such as hernia, granuloma, abscess, or neoplastic lesions [4,7,12]. In contrast, secondary abdominal wall endometriosis is typically related to prior surgical procedures, most commonly cesarean section, and occurs within or adjacent to the surgical scar due to direct implantation of endometrial tissue at the time of surgery [13].

The present case is noteworthy as it represents primary periumbilical endometriosis, diagnosed six years after a cesarean section and notably occurring in the absence of clinical, radiological, or intraoperative evidence of pelvic endometriosis. Although the patient had undergone a Pfannenstiel cesarean incision, the lesion was located at the umbilicus, anatomically distant from the surgical scar, and developed several years after the procedure. The absence of direct umbilical manipulation and the delayed presentation support a primary rather than a secondary etiology. A similar case was reported by Beyene et al. in which primary umbilical endometriosis developed nine years after a cesarean section and in the absence of any prior umbilical intervention, supporting that remote cesarean history alone does not necessarily indicate secondary implantation [14]. Primary umbilical endometriosis in the absence of direct umbilical surgery has been similarly documented in case series and literature reviews, reinforcing the need for clinical vigilance even in women with remote surgical histories [5,15].

The pathogenesis of primary umbilical endometriosis remains incompletely understood. The predominant theories include lymphatic or hematogenous dissemination of endometrial cells, coelomic metaplasia of primitive cells, and transformation involving embryonic remnants such as the urachus [4]. A systematic literature review demonstrated support for both lymphovascular spread and metaplastic transformation in patients lacking concomitant pelvic disease [16]. In the present case, the absence of pelvic endometriosis on contrast-enhanced MRI and the lack of intraoperative or surgical pathological evidence of pelvic lesions suggest a pathophysiological basis more consistent with distant cell migration or local metaplasia, rather than direct surgical implantation or contiguous extension.

Clinically, umbilical endometriosis most commonly presents as a firm umbilical or periumbilical nodule associated with cyclical pain, swelling, discoloration, or occasionally bleeding in synchronization with menstrual cycles [5,7]. In the present patient, cyclical periumbilical pain was the dominant presenting feature without continuous bleeding. This presentation aligns with the findings of Dridi et al. in their monocentric series of umbilical endometriosis cases, identifying pain as the most frequent presenting complaint, while bleeding and ulceration were comparatively uncommon [5]. The patient’s severe dysmenorrhea and menorrhagia were attributable to an incidental intramural uterine fibroid rather than pelvic endometriosis, making this a notable case of coexisting but pathophysiologically distinct gynecological pathology.

Imaging serves as an important adjunct in the evaluation but lacks pathognomonic, disease-specific radiologic features. Ultrasound typically demonstrates a nonspecific subcutaneous mass, and misclassification as an umbilical hernia is a frequently reported diagnostic pitfall [12,15,17]. In the present case, the initial ultrasound misinterpreted the periumbilical endometriotic lesion as an umbilical hernia, highlighting this diagnostic challenge. MRI, however, provides superior soft-tissue characterization and enables the exclusion of intraperitoneal or pelvic involvement. In our patient, the imaging pattern showed enhancing subcutaneous periumbilical lesions without any pelvic involvement, which is consistent with the imaging characteristics described in previous reports [7,8,12].

Accurate diagnosis requires histopathological confirmation following surgical excision. Wide local excision with clear margins remains the gold standard of treatment, offering excellent symptom resolution and low postoperative recurrence [5,8,11]. Total omphalectomy is often recommended for large, infiltrative, or recurrent lesions. However, for smaller nodules, umbilicus-sparing wide local excision is a reasonable approach, offering symptom resolution, as demonstrated in this case. In the present case, the excision performed during menstruation facilitated intraoperative identification due to characteristic lesion coloration, and the umbilicus was preserved for cosmetic reasons. Previous reports note that endometriosis nodules often become more painful, increase in size, and appear bluish or black during menstruation, which can improve their visibility during surgery [18,19]. At the six-month follow-up, the patient remained asymptomatic, which is consistent with the favorable outcomes reported in recent case series [8,11] and aligns with Hirata et al. [11], who also documented a low incidence of postoperative recurrence of umbilical lesions.

Hormonal therapies, including combined oral contraceptives, progestins, and gonadotropin-releasing hormone analogues, may provide temporary symptomatic relief but do not eradicate lesions and carry the risk of symptom recurrence after cessation [2,5]. As a result, medical management is generally reserved for patients who are not surgical candidates or who decline operative intervention.

This case report presents a rare instance of primary periumbilical/umbilical endometriosis supported by clear clinicoradiologic correlation and definitive histopathological confirmation following umbilicus‑sparing wide local excision, resulting in complete symptom resolution. The main limitation is that this is a single‑patient report with limited follow‑up, and although no pelvic involvement was identified clinically or radiologically, microscopic disease cannot be entirely excluded.

## Conclusions

This case underscores the importance of considering primary periumbilical endometriosis in women presenting with cyclical umbilical or periumbilical pain and nodules, even in the absence of direct umbilical surgery or pelvic disease. Early clinical suspicion, appropriate imaging, and complete surgical excision are essential to achieve a definitive diagnosis, prevent recurrence, and improve the patient's quality of life.
